# Materia Medica and Therapeutics, with Especial Reference to the Clinical Application of Drugs

**Published:** 1892-03

**Authors:** 


*Materia Medica and Therapeutics, with especial reference to the Clinical application of Drugs.* By JOHN V. SHOEMAKER, A.M., M.D. Vol. II. of a Treatise on Materia Medica, Pharmacology, and Therapeutics. Being an Independent Volume upon Drugs. Pp. xxi., 355—1004. Philadelphia: F. A. Davis. 1891.

This, the second volume of the work by Prof. Shoemaker, has appeared at a considerable interval after the first part, and in its preparation Dr. Aulde seems to have had no share, as he had in the former. The substances are arranged in alphabetical order, thus facilitating the process of finding the information afforded about any particular remedy; in the existing state of therapeutics this is the only sensible method to follow. The work deals comparatively slightly with the chemistry or even pharmacology of drugs, but treats of their therapeutical application with convenient fulness; very many useful formulæ and practical hints in the employment of remedies are given in the text, and will be of much use to practitioners. The delay in publishing the volume has enabled Prof. Shoemaker to bring the matter well up to date, so that there can be found full information upon such new remedial agents as tuberculin, spermine, exalgine, pyoktanin, and so forth. Besides these and such like, there receive description many substances practically unknown to us in England, such as hoang-nan, squaw-vine and Mormon tea. Taking the first and second volumes together, the book is undoubtedly the most far-reaching of works on therapeutics in the English language; from the point of view of physiology it is not in comparison with Lauder Brunton's book, and perhaps some others, but as a dictionary of remedies and their uses it is as yet the most complete. The index-maker has also done excellent work.

